# Comparative Validation of a VISULYZE‐Based Nomogram for Predicting Visual Outcomes and Higher‐Order Aberrations After SMILE in High Myopia

**DOI:** 10.1155/joph/1918446

**Published:** 2026-06-18

**Authors:** Lingling Zhao, Nannan Gao, Enze Liu, Xinliang Cheng, Juehan Li, Haixia Ji, Ying Yu

**Affiliations:** ^1^ Eye Institute, Affiliated Hospital of Nantong University, Medical School of Nantong University, Nantong, Jiangsu, China, ntu.edu.cn

**Keywords:** high myopia, higher-order aberrations, nomogram, small incision lenticule extraction, VISULYZE

## Abstract

**Purpose:**

To validate a novel VISULYZE‐based nomogram for high myopia in small incision lenticule extraction (SMILE) surgery.

**Methods:**

This prospective, nonrandomized controlled trial included 118 eyes of 71 high‐myopia patients treated with SMILE using a VisuMax 500‐kHz femtosecond laser. High myopia was defined as a spherical equivalent refraction ≤ −6.00 D. Treated eyes were divided into two groups, according to different nomograms received: the conventional surgeon‐adjusted nomogram group (*n* = 71 eyes) and the VISULYZE‐based nomogram group (*n* = 47 eyes). The VISULYZE nomogram was generated by the data from 108 myopic eyes that underwent SMILE surgery at 3 months. The visual outcomes followed up for 3 months for both groups were compared for efficacy, safety, predictability, stability, and higher‐order aberrations (HOAs).

**Results:**

Preop‐SE was −6.58 ± 0.50 D in the conventional group and −6.79 ± 0.61 D in the VISULYZE group. At 3 months postoperatively, the SE in the conventional group and the VISULYZE group was −0.16 ± 0.34 D and 0.05 ± 0.40 D (*p* < 0.05), respectively. It was found that 72% and 100% had a UDVA of 20/20 in the conventional group and VISULYZE group, respectively, while 89% and 99% had an SE within ±0.5 D and ±1.0 D in the conventional group and 89% and 100% in the VISULYZE group. The efficacy indexes were 0.97 ± 0.07 in the conventional group and 1.02 ± 0.06 in the VISULYZE group (*p* < 0.05). The safety indexes were 1.15 ± 0.10 in the conventional group and 1.15 ± 0.15 in the VISULYZE group (*p* > 0.05), respectively. The VISULYZE‐based nomogram was associated with lower induction of 3rd‐order vertical/horizontal coma, total coma, 4th‐order spherical aberration, and root mean square (RMS) HOAs.

**Conclusions:**

The VISULYZE‐based nomogram demonstrated comparable safety to the conventional nomogram while achieving significantly superior efficacy and predictability. Additionally, it provided a supportive condition for reducing RMS HOAs after SMILE compared to the conventional approach.

**Trial Registration:** ClinicalTrials.gov: NCT06982807

## 1. Introduction

By 2050, high myopia is projected to affect 938 million people globally, representing 9.8% of the world population [1]. In 2025, the Chinese Center for Disease Control and Prevention reported that over 70 million people in China were affected by high myopia, representing 43% of the global disease burden.

With its no‐flap and all‐in‐one laser procedure, small incision lenticule extraction (SMILE) surgery has been used worldwide. Extensive clinical evidence has established the safety, efficacy, and long‐term stability of SMILE surgery in correcting high myopia since its introduction [2–4]. However, postoperative overcorrection and undercorrection remain clinical challenges; for example, Jin et al. [5] reported that compared with mild to moderate myopia, the SE in high myopia showed undercorrection in SMILE. Ramin et al. [6] showed that overcorrection in eyes with high myopia produced a higher frequency of myopia regression.

Surgical nomograms have consequently been developed as evidence‐based tools to enhance refractive predictability and optimize patient outcomes [7]. Now, nomogram adjustment has been employed in the SMILE procedure to improve refractive outcomes for myopic correction [8]. However, a standardized nomogram for myopia correction has yet to be established, despite multiple proposed methodologies in the refractive surgery literature. Furthermore, interpatient variability in ocular biometry, combined with differences in surgical technique, clinician experience, and laser platform specifications, may account for deviations in postoperative visual outcomes [9].

VISULYZE is an integrated software platform for clinical data analysis and personalized nomogram generation. With a simple and intuitive interface, it provides a clear overview of the clinical results and generates nomograms customized to each user. But the clinical efficacy of this system remains unreported in peer‐reviewed literature. The purpose of this study is to comprehensively compare the refractive efficacy, safety, predictability, stability, and higher‐order aberrations (HOAs) predicted by the VISULYZE‐based nomogram with the conventional surgeon‐adjusted nomogram after SMILE for high myopia. For brevity, the conventional surgeon‐adjusted nomogram group and the VISULYZE‐generated nomogram group are referred to as the conventional group and VISULYZE group, respectively.

## 2. Patients and Methods

### 2.1. Patients

This prospective, nonrandomized controlled trial (RCT) was conducted in accordance with the Declaration of Helsinki, approved by the Ethics Committee of the Affiliated Hospital of Nantong University. Patients were recruited from November 2024 to March 2025 at the Affiliated Hospital of Nantong University and were divided into two groups according to the received nomograms.

All patients who were enrolled in this study underwent a comprehensive preoperative ophthalmologic examination, which was always performed by the same team of professional ophthalmologists and optometrists, including uncorrected distance visual acuity (UDVA), corrected‐distance visual acuity (CDVA), manifest refraction, slit‐lamp examination, dilated fundus examination, and measurements of corneal topography (Pentacam HR Oculus, Wetzlar, Germany).

The inclusion criteria were as follows: (1) aged more than 18 years; (2) stable refraction error in the preceding 2 years; (3) sufficient residual corneal bed thickness more than 280 μm after SMILE; (4) previous spherical equivalent (SE) more than −6.00 D; and (5) absence of soft contact lenses for at least 2 weeks, hard contact lenses for 1 month, and Ortho‐K contact lenses for 3 months. The exclusion criteria were as follows: (1) a history of other corneal trauma or diseases; (2) a history of systemic diseases; (3) women in pregnancy or lactation; and (4) suspicion of keratoconus on corneal topography.

### 2.2. Nomogram Setting

#### 2.2.1. Conventional Surgeon‐Adjusted Nomogram

Adjusting slightly according to age, sex, accommodation status, surgeon’s experience, and hospital environment, the nomogram was derived from the formula as follows: SE error = 0.25 + 0.10 × SE_preoperative_ [10, 11].

#### 2.2.2. VISULYZE‐Based Nomogram

**FIGURE 1:**
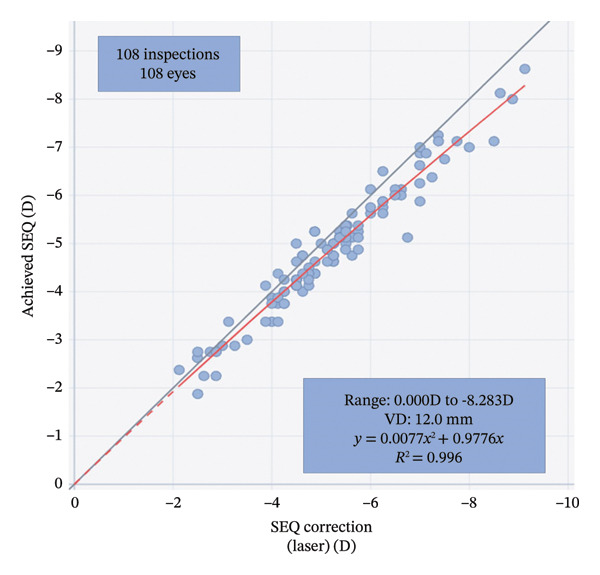
Nomogram generated by VISULYZE; SEQ, spherical equivalent refraction; D, diopters.

The nomogram of the VISULYZE group was generated by the VISULYZE platform (Carl Zeiss Meditec AG), which employs artificial intelligence (AI) and big data analytics to optimize surgical parameters through retrospective case analysis. As a comprehensive data analysis and personalized nomogram generation tool, VISULYZE is designed to eliminate four types of systematic errors (differences in refractometry equipment, optometrists’ habits, surgical devices, and surgeon techniques) by scientifically analyzing historical surgical data from the same center, same surgeon, and same device, thereby generating a dedicated personalized calibration value with a correction accuracy of up to 0.001 D. According to the software instructions, we retrospectively collected and input the refractive data, including age, gender, eye (OD/OS), clinical target, and the device type, as well as preoperative and 3 months postoperative spherical power (SPH), cylindrical power (CYL), axis position (AX), UDVA, and CDVA from 108 eye samples of surgical patients who previously underwent SMILE surgery at the Affiliated Hospital of Nantong University from October 2022 to August 2024. The generated nomogram is as shown in Figure 1.

### 2.3. Surgical Techniques

In the study, all surgical procedures were performed under topical anesthesia by the same experienced surgeon using the VisuMax 500‐kHz femtosecond laser (Carl Zeiss Meditec AG, Jena, Germany). The surgery parameters were set as follows: Cap thickness was between 110 and 120 μm, and cap diameter was 7.2–7.5 mm. The optical zone varied between 6.2 and 6.5 mm. A superior corneal incision (2 mm length, 11–12 o’clock position) was created, followed by manual lenticule extraction.

The surgeon had over 15 years of clinical experience in SMILE surgery and was proficient in manual centration using the VisuMax 500 platform. Preoperatively, patients with high astigmatism (the cylindrical refraction > −2.00 D) underwent limbal marking at the 3 and 9 o’clock positions along the horizontal meridian under the slit lamp. During surgery, gentle rotation of the cone was performed to align the limbal marks with the horizontal axis of the microscope eyepiece reticule, thereby achieving effective compensation for cyclotorsion.

### 2.4. Postoperative Evaluation

Follow‐up appointments were scheduled at 10 days, 1 month, and 3 months after surgery. Postoperative examinations included slit‐lamp examination, measurements of UDVA, CDVA, and Pentacam imaging examinations. Vertical and horizontal comas, vertical and oblique trefoil, spherical aberration, and the root mean square (RMS) of total HOAs measured over the entire cornea were used in this study.

### 2.5. Statistical Analysis

The statistical analysis was conducted using SPSS software (Version 27.0, IBM Corporation). The Kolmogorov–Smirnov (K–S) test was employed to assess the normality of data distributions, guiding the choice between parametric and nonparametric tests. The independent‐samples *t* test was used to compare data in normal distribution. When the data did not fit normal distribution, the Mann–Whitney test was used for comparison. Qualitative or categorical data were analyzed by the chi‐square test. A paired‐sample *t* test and ANOVA test were used for preoperative and postoperative comparisons. Accounting for inter‐eye variability, data from both eyes were analyzed simultaneously using a generalized estimating equation (GEE) to ensure accuracy in both groups. Multivariate linear regression (LR) (enter method) was used to assess the independent association between ΔHOA and age, preoperative HOAs, group, and ΔSE. The variance inflation factor (VIF) was checked for multicollinearity. Continuous values are presented as the mean ± standard deviation (SD). A *p* value less than 0.05 was considered statistically significant.

## 3. Results

Of the 125 eye samples from 75 patients, 4 eye samples (2 patients) were lost from the conventional group, and 3 eye samples (2 patients) were lost from the VISULYZE group. A total of 71 eye samples (41 patients) from the conventional group and 47 eye samples (30 patients) from the VISULYZE group were used for evaluation. Table 1 shows the statistical analysis of eye samples from each group. No significant difference between the conventional group and the VISULYZE groups preoperatively.

**TABLE 1:** Demographic and preoperative patient information (mean ± SD and range).

Characteristics	Conventional group	VISULYZE group	*p* value
Eye (OD/OS)	38/33	26/21	0.612
Gender (M/F)	20/21	18/12	0.349
Age (years)	25.22 ± 4.85 (19∼35)	25.30 ± 6.59 (18∼45)	0.678
IOP (mmHg)	11.90 ± 2.51 (8∼17)	11.64 ± 1.98 (7∼16)	0.223
Sphere (D)	−5.93 ± 0.66 (−4.75∼−8.25)	−6.21 ± 0.79 (−5.00∼−8.25)	0.443
Cylinder (D)	−1.28 ± 0.80 (0∼−3.00)	−1.16 ± 0.88 (0∼−3.25)	0.146
SE (D)	−6.58 ± 0.50 (−6.00∼−8.25)	−6.79 ± 0.61 (−6.00∼−8.25)	0.979

*Note:* M, male; F, female; IOP, intraocular pressure; D, diopters.

Abbreviations: SD, standard deviation; SE, spherical equivalent.

### 3.1. Efficacy and Safety

**FIGURE 2:**
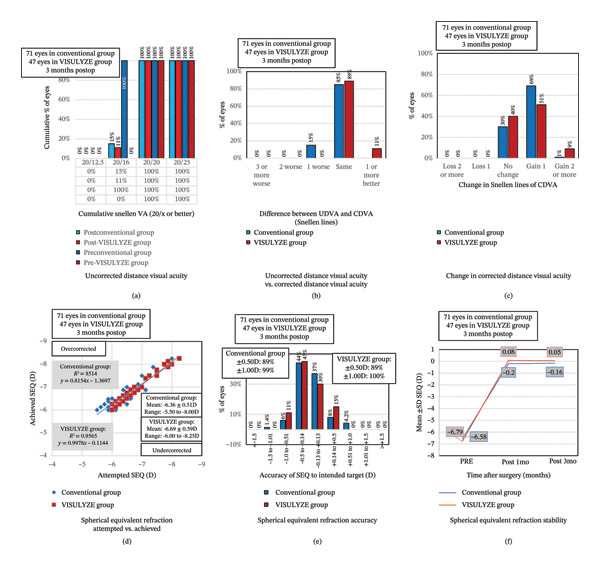
Standard graphs of refractive surgery showed the visual and refractive outcomes in the conventional group and VISULYZE group at 3‐month follow‐up. UDVA, uncorrected distance visual acuity; CDVA, corrected distance visual acuity; D, diopters; Pre, preoperative; Post, postoperative; SEQ, spherical equivalent refraction.

The comparisons of UDVA and CDVA after surgery between the two groups are shown in Figure 2a, which illustrates the efficacy of the two groups by cumulative percentage of preoperative CDVA and postoperative UDVA at 3 months’ follow‐up. In total, 72% (51/71) and 100% (47/47) reached 20/20 or better at the 3‐month follow‐up in the conventional group and VISULYZE group, respectively. As shown in Figure 2b, at 3 months postoperatively, 15% (11/71) of eyes in the conventional group had a UDVA of 1 line worse, while 11% (5/47) of eyes in the VISULYZE group had a UDVA of 1 or more lines better. 85% (60/71) and 89% (42/47) had the same UDVA. The efficacy indexes at 3 months were 0.97 ± 0.07 in the conventional group and 1.02 ± 0.06 in the VISULYZE group, respectively. There existed a significant difference between the two groups (*p* < 0.05).

Safety is illustrated in Figure 2c; postoperatively, no one lost one line of CDVA in either SMILE group. 69% (49/71) and 51% (24/47) gained one line of CDVA, 1% (1/71) and 9% (4/47) gained 2 lines of CDVA, and there was no change in 30% (21/71) and 40% (19/47) in the conventional group and VISULYZE group, respectively. The safety indexes at 3 months were 1.15 ± 0.10 in the conventional group and 1.15 ± 0.15 in the VISULYZE group. There was no significant difference between the two groups (*p* > 0.05).

### 3.2. Predictability and Stability

Figure 2d shows a scatterplot and LR analysis of attempted versus achieved spherical equivalent refraction at 3 months after surgery, which indicates that the VISULYZE group (*R* ^2^ = 0.9565) had better performance than the Conventional group (*R* ^2^ = 0.8514). Figure 2e illustrated the predictability of the surgery in both groups. At 3 months, 89% (63 eyes) and 99% (70 eyes) were within ±0.5 D and ±1.0 D of the intended correction in the conventional group, and 89% (42 eyes) and 100% (47 eyes) were within ±0.5 D and ±1.0 D in the VISULYZE group, respectively. Figure 2f shows the mean postoperative SE of the two groups. At 3 months postoperatively, the SE in the conventional group and VISULYZE group was −0.16 ± 0.34 D and 0.05 ± 0.40 D (*p* < 0.05). Significant differences were also found between the two groups at 1 month after surgery (*p* < 0.05).

### 3.3. Higher‐Order Aberrations

There was no significant difference in HOAs between the conventional group and the VISULYZE groups preoperatively. Postoperatively, at 3 months, the conventional group showed significant increases in vertical coma, total coma, trefoil, spherical aberration, and total RMS HOA, whereas the VISULYZE group exhibited significant increases only in vertical coma, total coma, and RMS HOA. The increases of vertical coma, coma, spherical aberration and RMS HOA were lower in the VISULYZE group than in the conventional group. Table 2 shows the comparison of aberrations before and after SMILE surgery between the conventional group and the VISULYZE group.

**TABLE 2:** Comparison of aberration before and after SMILE surgery between the conventional group and the VISULYZE group (mean ± SD).

Time	Pre‐op			Post‐3M		
Group	Conventional group	VISULYZE group	*p*	Conventional group	VISULYZE group	*p*
Vertical coma Z (3, −1)	−0.09 ± 0.29^#^	0.03 ± 0.18^$^	0.514	−0.67 ± 0.37^#^	−0.30 ± 0.20^$^	< 0.001^∗^
Horizontal coma Z (3, 1)	−0.01 ± 0.16	−0.02 ± 0.14	0.425	0.03 ± 0.30	0.04 ± 0.21	0.856
Coma	0.28 ± 0.19^#^	0.20 ± 0.12^$^	0.293	0.76 ± 0.32^#^	0.38 ± 0.17^$^	< 0.001^∗^
Vertical trefoil Z (3, −3)	−0.04 ± 0.11	−0.07 ± 0.13	0.224	−0.04 ± 0.14	−0.05 ± 0.15	0.880
Oblique trefoil Z (3, 3)	0.01 ± 0.11	−0.03 ± 0.12	0.142	−0.01 ± 0.13	0.01 ± 0.14	0.620
Trefoil	0.13 ± 0.09^#^	0.16 ± 0.10	0.104	0.17 ± 0.09^#^	0.19 ± 0.10	0.390
Spherical aberration Z (4, 0)	0.24 ± 0.14^#^	0.20 ± 0.06	0.600	0.44 ± 0.17^#^	0.24 ± 0.17	< 0.001^∗^
RMS HOA	0.48 ± 0.21^#^	0.40 ± 0.10^$^	0.214	0.98 ± 0.26^#^	0.68 ± 0.13^$^	0.000^∗^

*Note:* SMILE, small incision lenticule extraction; Pre‐op, preoperative; Post, postoperative; 3M, 3 months.

Abbreviations: HOA, higher‐order aberration; RMS, root mean square.

^#^Significant difference in HOAs at post‐3M compared with preoperatively (*p* < 0.05) in the conventional group.

^$^Significant difference in HOAs at post‐3M compared with preoperatively (*p* < 0.05) in the VISULYZE group.

^∗^Significant difference in HOAs at 3 months between two groups (*p* < 0.05).

As shown in Table 3, the analysis of changes in HOAs between preoperative and 3‐month postoperative measurements revealed that the VISULYZE group had significantly smaller differences in 3rd‐order vertical coma, total coma, 4th‐order spherical aberration, and total RMS HOAs compared to the conventional group.

**TABLE 3:** Amount of induced higher‐order aberrations (HOAs) of SMILEs using different nomograms.

	**Z (3, −1)**	**Z (3, 1)**	**Coma**	**Z (3, −3)**	**Z (3, 3)**	**Trefoil**	**Z (4, 0)**	**RMS HOA**

Conventional group	−0.58	0.04	0.48	0.00	−0.02	0.04	0.20	0.50
VISULYZE group	−0.33	0.06	0.18	0.02	0.04	0.03	0.04	0.28
*p* value	< 0.001^∗^	0.940	< 0.001^∗^	0.556	0.121	0.702	< 0.001^∗^	< 0.001^∗^

*Note:* The total amount of induced HOAs was determined by subtracting the preoperative HOAs values from the 3‐month postoperative HOAs values. SMILE, small incision lenticule extraction.

Abbreviations: HOA, higher‐order aberration; RMS, root mean square.

^∗^ *p* < 0.05.

**FIGURE 3:**
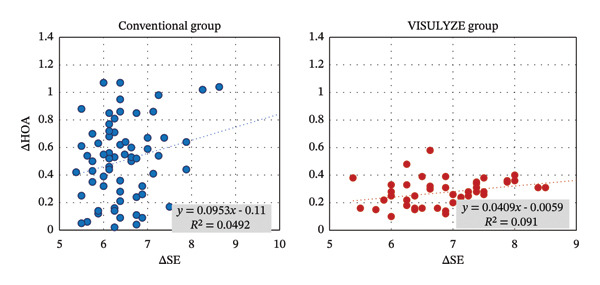
Correlation and regression analysis of ΔHOA versus ΔSE. Each scatter plot with a linear regression line shows the equation of the regression line and the corresponding *R* ^2^ value.

Furthermore, the correlation and regression between the preoperative and postoperative differences in ΔHOA and ΔSE was analyzed. As shown in Figure 3, with increasing ΔSE, the ΔHOA increased less in the VISULYZE group than in the Conventional group after adjustment with the VISULYZE nomogram. To further investigate the independent association between ΔSE and ΔHOA, multivariate LR analysis was performed with ΔHOA as the dependent variable and age, preop‐HOAs, group, and ΔSE as independent variables. As shown in Table 4, after adjusting for age and preop‐HOAs, ΔSE remained significantly associated with ΔHOA (*p* = 0.005), indicating that a smaller residual refractive error is associated with lower induced HOAs. Notably, the group variable also remained highly significant (*p* < 0.001) with a larger standardized coefficient than that of ΔSE. These results suggest that the lower HOAs observed in the VISULYZE group are not fully explained by improved refractive accuracy alone. However, as VISULYZE is a preoperative planning tool and does not directly control intraoperative centration or cyclotorsion, and because intraoperative centration parameters were not measured in this study, the observed statistical association should be interpreted as a supportive condition rather than a direct causal mechanism. The platform may be associated with lower HOAs, possibly by reducing systematic errors in the preoperative workflow, but the exact pathway remains unclear and requires further investigation.

**TABLE 4:** Multivariate linear regression analysis of association between clinical parameters and ΔHOA after SMILE surgery.

Parameters	*B* (95% CI)	Beta	*t*	*p*
Age (years)	0.000 (−0.007, 0.006)	−0.006	−0.085	0.932
Preop‐HOAs	−0.568 (−0.782, −0.353)	−0.396	−5.247	< 0.001^∗^
Group	−0.302 (−0.382, −0.222)	−0.589	−7.452	< 0.001^∗^
ΔSE (D)	0.079 (0.025, 0.133)	0.223	2.877	0.005^∗^

*Note:* ΔHOA, change in total higher‐order aberrations (RMS); ΔSE, change in spherical equivalent; Preop‐HOAs, preoperative HOAs. Adjusted *R* ^2^ = 0.362, model: *F* (4, 113) = 17.589.

Abbreviation: CI, confidence interval.

^∗^ *p* < 0.05.

## 4. Discussion

Previous studies have established nomograms for adjusting SE and astigmatism for LASIK [12, 13]. Recently, more attention has been focused on nomograms for correcting refractive errors in SMILE procedures. Various nomogram designs for myopia correction may improve the predictability of surgery. SMILE nomogram adjustment is largely based on surgeons’ clinical experience. In 2017, Liang et al. [11] first systematically validated the optimizing effect of the nomogram in SMILE surgery, establishing a linear correction formula (SE error = 0.259 + 0.113 × SE_preoperative_) through multiple regression analysis. Without compromising safety and efficiency, the study increased the ±0.50 D predictability from 70% to 86% and the ±1.00 D predictability from 97% to 98%. However, given patient diversity, the nomogram used in the study may not be directly applicable to other surgeons. With the accumulation of data and the improvement of computational power, Cui et al. [14] adopted a machine learning (ML) technique to develop nomogram models for SMILE. These ML‐based models demonstrated comparable safety profiles to surgeons while achieving significantly superior efficacy. In terms of predictability, the ML group achieved a higher proportion of postoperative refractive errors within ±0.50 D (93%) compared to the surgeon group (83%), demonstrated a stronger linear correlation (*R* ^2^ = 0.9645 vs. 0.9528), and had a significantly better mean SE correction error (−0.09 D vs. −0.23 D). However, due to the lack of eye samples, the ML‐based nomograms have limitations in correcting high myopia and astigmatism. With the application of AI in medical fields, Park et al. [15] proposed an AI‐based approach to develop nomograms for SMILE, including various machine learning algorithms: multiple LR, decision tree, AdaBoost, XGBoost, and multilayer perceptron (MLP). The study proved the feasibility of applying AI to nomograms for SMILE. Building on ML‐based nomograms, Luft et al. [16] subsequently developed three AI‐driven models: a linear model (LM), a generalized additive mixed model (GAMM), and an artificial neural network (ANN). Adjustment using LM, GAMM, and ANN reduced the mean refractive error toward zero and decreased variance, though ANN showed slight overcorrection. Clinically, all models marginally increased the proportion of eyes within ±0.50D of targeted SE (LM: 83%, GAMM: 84%, ANN: 83% vs. baseline 82%). Furthermore, Liu et al. [17] employed XGBoost, gradient boosting regression (GBR), random forest (RF), LightGBM, LR, and support vector regression (SVR), which are machine learning techniques, to develop the nomogram prediction model, which could accurately predict the nomogram for SMILE. However, a standardized nomogram for myopia correction has yet to be established. In our study, we created personalized nomograms utilizing the VISULYZE platform in accordance with the manufacturer’s guidelines. We then compared 3‐month postoperative outcomes between the conventional nomogram (conventional group) and the VISULYZE‐based nomogram (VISULYZE group), with particular focus on changes in HOAs. Our study shows that the VISULYZE group achieved significantly better refractive efficacy and predictability for high myopia correction than the conventional group, while maintaining safety.

Research has shown that in SMILE surgery, the induction of HOAs is associated with the degree of myopia [18, 19]. Our multivariate regression analysis further clarified the relationship between refractive accuracy and HOAs. Although ΔSE was significantly associated with ΔHOA (*p* = 0.005), the group effect remained highly significant (*p* < 0.001) with a larger standardized coefficient, even after controlling for ΔSE. Despite the inevitable induction of HOAs in high myopia [20], the VISULYZE‐based nomogram was associated with lower HOAs than the traditional approach. Notably, the clinical outcomes of VisuMax 500, especially HOAs and astigmatic correction, are closely related to the surgeon’s manual skill in centration and cyclotorsion compensation [21, 22]. To minimize possible confounding effects, several rigorous strategies were adopted in the present study: All procedures were performed by the same experienced surgeon who had received standardized operation training, including preoperative limbal horizontal marking and intraoperative adjustment for cyclotorsion compensation, and identical surgical parameters were applied across both groups. It is important to clarify that the VISULYZE platform itself is a preoperative nomogram generation tool and does not possess built‐in functions for real‐time intraoperative centration or cyclotorsion adjustment. The management of centration‐refraction interaction relies on the hardware capabilities of the surgical platform rather than the VISULYZE software. In the present study using the VisuMax 500 platform, centration and cyclotorsion compensation were achieved through the rigorous manual techniques. Moreover, recent studies have demonstrated that VisuMax 800, equipped with CentraLign and OcuLign, induces fewer HOAs compared to VisuMax 500, which relies on manual operation [23]. Future studies could therefore explore the integration of the VISULYZE nomogram with the VisuMax 800 platform to further reduce postoperative HOAs and provide more optimized surgical options for clinical practice.

Traditional nomograms are primarily based on surgeons’ empirical experience, gradually refined through accumulated surgical data. In contrast, the VISULYZE system has further significantly advanced nomogram development through a more scientific and systematic approach, utilizing big data analytics and AI technologies. Beyond incorporating sophisticated AI algorithms, VISULYZE places particular emphasis on retrospective analysis of postoperative outcomes, leveraging these data‐driven insights to optimize personalized surgical planning. With VISULYZE, doctors can create visual tools such as the refractive nine‐graph and astigmatism polar plot, which intuitively demonstrate the surgery’s effectiveness, safety, predictability, and stability. The refractive nine‐graph illustrates the distribution of spherical and astigmatic corrections, aiding doctors in quickly identifying issues and optimizing surgical plans. Moreover, by analyzing outcomes from previous patients, it enhances surgical planning for future cases. In summary, VISULYZE not only assists doctors in addressing surgical variations but also ensures clearer and more stable postoperative visual outcomes for patients. Recent studies have demonstrated the feasibility of “codeless development” of customized SMILE nomograms using large language models (LLMs) represented by ChatGPT‐4. This approach offers a practical and accessible framework for clinicians to achieve personalized nomogram customization without reliance on proprietary software such as VISULYZE [24]. The LLM‐derived nomogram exhibited high consistency with traditional statistical software (R and SPSS), with Pearson correlation coefficients of 0.993 for sphere prediction and 0.979 for cylinder prediction. The mean absolute error (MAE) of spherical equivalent prediction was 0.2696 D, which was comparable to that of R (0.2691 D) and SPSS (0.2699 D) and significantly superior to the simple empirical nomogram (0.4352 D). Additionally, ChatGPT‐4 was capable of automatically generating HTML code for a web‐based calculator, enabling real‐time output of customized surgical parameters upon input of preoperative data. However, Jun’s work was retrospective, and prospective clinical trials are still lacking to verify the long‐term predictive stability and generalizability of LLM‐derived nomograms across diverse regions and patient populations. Moreover, frontline clinicians may face implementation barriers due to insufficient familiarity with AI tool operation and prompt design, hindering grassroots application. In the future, in‐depth integration of LLM‐driven big data analytics with clinically validated, mature algorithms from VISULYZE holds great promise. Specifically, LLMs can first mine massive clinical datasets to identify potential complex correlations and novel predictive factors, and the derived data insights will then undergo precise modeling and rigorous validation through VISULYZE’s well‐established algorithmic framework. This integrated approach—combining data‐driven discovery with algorithm‐driven validation—ensures the clinical applicability and stability of the resulting nomogram, not only enhancing the predictive accuracy and personalization of nomograms but also laying a solid foundation for their widespread translation and application across diverse clinical scenarios.

While random selection of one eye per patient mitigates inter‐eye bias, evidence suggests eye laterality (right/left) may influence postoperative refractive outcomes and subsequent nomogram adjustment [7, 14, 25]. To improve the predictive accuracy of the machine learning models for nomogram development, we therefore utilized data from both eyes, and the GEE formula was employed to rectify inter‐eye variability. There are several limitations in the present study. The small sample size of SMILE cases may raise concerns about the statistical power of comparative analyses. A larger multicenter study is needed to confirm our findings. Moreover, single‐center data often exhibit a high degree of consistency in surgical procedures, equipment parameters, postoperative follow‐up protocols, and patient demographics. If the same nomogram were applied directly to a multicenter setting, the predictive accuracy (*R* ^2^) might decrease due to increased heterogeneity in devices, patient characteristics, and practice patterns. However, such a decrease would not invalidate our single‐center findings. Rather, it highlights the importance of developing and validating predictive models in the specific clinical context where they will be deployed. Accordingly, the nomogram developed in this study is intended for use within our center, and its performance in other settings should be evaluated separately before external application. We fully acknowledge that the generalizability of this model to other centers or other surgeon populations needs further validation, a limitation shared by all single‐center modeling studies. Future multicenter external validation is needed to confirm the cross‐center stability of the model. Moreover, this study only had a 3‐month follow‐up period, long‐term follow‐up is essential. Additional analyses such as subgroup analysis according to preoperative SE or attempted correction, outlier sensitivity, and centration assessment could not be performed due to the absence of intraoperative centration data, limited sample size, and lack of formal outlier analysis, although no extreme values were observed; these limitations are acknowledged, and future studies with larger samples and centration‐controlled platforms are needed. In addition, this study is a prospective non‐RCT. Although we have adopted a series of rigorous measures to reduce the potential risk of baseline imbalance and selection bias: all enrolled patients completed a comprehensive preoperative ophthalmologic examination conducted uniformly by the same team of professional ophthalmologists and optometrists; statistical analysis verified no significant differences in all preoperative clinical indicators, including spherical equivalent refraction, corneal biometrics, and HOAs, between the Conventional group and VISULYZE groups; and the GEE was applied for statistical analysis of bilateral eye data to correct the potential bias caused by non‐independent samples, this study cannot completely exclude the potential risk of baseline imbalance or selection bias. Future studies will adopt a RCT design with larger sample sizes and multicenter collaboration to further control potential biases and validate the conclusions of this study.

## 5. Conclusion

This study validated a VISULYZE‐based nomogram for high myopia correction. The nomogram demonstrated superior efficacy and predictability and was associated with fewer measured HOAs, indicating that the VISULYZE‐based approach can create favorable conditions for reducing HOAs after SMILE. These results demonstrate VISULYZE’s clinical value for creating personalized nomograms that improve both the precision and visual outcomes of SMILE surgery.

## Author Contributions

The authors confirm contributions to the manuscript as follows: conception and design of study: Ying Yu and Haixia Ji; data collection: Enze Liu, Xinliang Cheng, and Juehan Li; analysis and interpretation of results: Lingling Zhao and Nannan Gao; drafting the manuscript: Lingling Zhao and Nannan Gao.

## Funding

This project has been funded in part by grants from the Jiangsu Health International Exchange Support Plan and Jiangsu Provincial Research Hospital (YJXYY202204).

## Disclosure

All authors reviewed the results and approved the final version of the manuscript.

## Ethics Statement

The authors confirm that any aspect of the work covered in this manuscript that involved human patients was conducted with the ethical approval of all relevant bodies, and the study was performed in accordance with the Declaration of Helsinki. The protocol was approved by the Ethics Committee of the Affiliated Hospital of Nantong University. Signed informed consent documentation was obtained from the individual participants for their participation in the study.

## Conflicts of Interest

The authors declare no conflicts of interest.

## Data Availability

The data that support the findings of this study are available on request from the corresponding authors. The data are not publicly available due to privacy or ethical restrictions.
